# Targeting Vascular Endothelial Growth Factor Receptor 2 and Protein Kinase D1 Related Pathways by a Multiple Kinase Inhibitor in Angiogenesis and Inflammation Related Processes *In Vitro*

**DOI:** 10.1371/journal.pone.0124234

**Published:** 2015-04-14

**Authors:** Attila Varga, Pál Gyulavári, Zoltán Greff, Krisztina Futosi, Tamás Németh, Laura Simon-Szabó, Krisztina Kerekes, Csaba Szántai-Kis, Diána Brauswetter, Márton Kokas, Gábor Borbély, Anna Erdei, Attila Mócsai, György Kéri, Tibor Vántus

**Affiliations:** 1 Pathobiochemistry Research Group, Hungarian Academy of Sciences—Semmelweis University, Budapest, Hungary; 2 Vichem Chemie Research Ltd., Budapest, Hungary; 3 Department of Physiology, Semmelweis University, Budapest, Hungary; 4 Department of Immunology, Eötvös Loránd University, Budapest, Hungary; 5 Department of Medical Chemistry, Molecular Biology and Pathobiochemistry, Semmelweis University, Budapest, Hungary; Medical College of Wisconsin, UNITED STATES

## Abstract

Emerging evidence suggests that the vascular endothelial growth factor receptor 2 (VEGFR2) and protein kinase D1 (PKD1) signaling axis plays a critical role in normal and pathological angiogenesis and inflammation related processes. Despite all efforts, the currently available therapeutic interventions are limited. Prior studies have also proved that a multiple target inhibitor can be more efficient compared to a single target one. Therefore, development of novel inflammatory pathway-specific inhibitors would be of great value. To test this possibility, we screened our molecular library using recombinant kinase assays and identified the previously described compound VCC251801 with strong inhibitory effect on both VEGFR2 and PKD1. We further analyzed the effect of VCC251801 in the endothelium-derived EA.hy926 cell line and in different inflammatory cell types. In EA.hy926 cells, VCC251801 potently inhibited the intracellular activation and signaling of VEGFR2 and PKD1 which inhibition eventually resulted in diminished cell proliferation. In this model, our compound was also an efficient inhibitor of *in vitro* angiogenesis by interfering with endothelial cell migration and tube formation processes. Our results from functional assays in inflammatory cellular models such as neutrophils and mast cells suggested an anti-inflammatory effect of VCC251801. The neutrophil study showed that VCC251801 specifically blocked the immobilized immune-complex and the adhesion dependent TNF-α -fibrinogen stimulated neutrophil activation. Furthermore, similar results were found in mast cell degranulation assay where VCC251801 caused significant reduction of mast cell response. In summary, we described a novel function of a multiple kinase inhibitor which strongly inhibits the VEGFR2-PKD1 signaling and might be a novel inhibitor of pathological inflammatory pathways.

## Introduction

In many pathological disorders angiogenesis and chronic inflammation occur together, for instance in rheumatoid arthritis and in cancer. Amongst many immune cells, e.g. neutrophils, basophils and mast cells play an important role in promoting pathological angiogenesis and the continuous recruitment of inflammatory cells which can also result in severe tissue damage [1–3].

Angiogenesis, the formation of new capillaries from an existing blood vessel, has an essential role during embryonic development, in adult life and in numerous pathological conditions such as severe inflammatory diseases, cancer growth and metastasis [4]. Amongst the known angiogenic factors the dominant regulator of normal and pathological angiogenesis is VEGF and the VEGFR signaling pathway. VEGFR tyrosine kinases consist of three known isoforms: VEGFR1, VEGFR2 and VEGFR3. VEGFR1 negatively regulates vasculogenesis during embryonic development, but it stimulates endothelial cell proliferation. VEGFR2 is essential in embryonic vasculogenesis and it is the dominant regulator of pathological angiogenesis as well. It triggers endothelial cell proliferation, migration, tubule formation, vascular permeability and it is also involved in several inflammatory processes [5]. Although VEGFR3 is not expressed by vascular endothelial cells, it is involved in the regulation of lymphangiogenesis [4].

PKD1 is a member of the protein kinase D family of serine/threonine kinases. Based on sequence homology of the kinase domains, PKDs are considered as Ca^2+^/calmodulin mediated kinases (CAMKs). The PKD family comprises three known members: PKD1 or PKCμ, PKD2 and PKD3 or PKCν [6–9]. The most well-characterized isoform is PKD1, which is involved in several physiological processes, such as oxidative stress response, cell motility and also in several pathological processes, such as cardiac hypertrophy, tumor development and tumor angiogenesis [10–13] [14]. In tumor angiogenesis, endothelial PKD1 has a positive regulatory function as the part of the VEGFR2 signaling pathway [15–19]. According to recent studies, VEGF activated PKD1 causes an inactivating phosphorylation on histone deacetylase 5 (HDAC5) and induces its nuclear exclusion and the induction of angiogenic gene expression [15,20]. In addition, PKD1 is involved in different inflammatory processes, for instance in neutrophils as the part of the Fcγ receptor signaling pathway it participates in the activation of NADPH-oxidase, which results in superoxide production. Furthermore in mast cells, macrophages, neutrophils, lung epithelial cells and endothelial cells the production of different inflammatory cytokines also requires PKD1 activation [21–24]. The pathological dysfunction of these cells and processes can be observed in numerous inflammatory diseases for example rheumatoid arthritis, sepsis and atherosclerosis [2].

In the last few years the paradigm of drug discovery changed from the single target drug to the multiple target drug approach [25]. Since in most tumors multiple signaling pathways are deregulated, small molecular inhibitors in future therapeutic strategies should be designed to target multiple signaling effectors and pathways. Using combination therapy, the major possibilities of inhibiting multiple targets are the simultaneous application of more than one drugs or a multiple target drug alone. The latter can be the solution for future medical applications, already with promising results in certain anticancer therapies [26]. Additionally, Hanahan and Weinberg highlighted tumor angiogenesis as a very special therapeutic target since anti-angiogenic therapy spares normal tissues better [27,28].

Our laboratory has several years of experience in kinase inhibitor development, especially regarding anti-inflammatory compounds (K.F., T.V., G.K., and A.M., unpublished observations, December 2010). Recognizing the low number of specific PKD inhibitors, we decided to search for novel multiple, angiogenesis and inflammation pathway inhibitors targeting VEGFR2 and PKD1.

As a result, we identified and characterized a multiple kinase inhibitor targeting VEGFR2 and PKD1, what effectively interferes with angiogenic and inflammation related processes in various cellular models.

## Materials and Methods

### Materials

Axitinib was obtained from M/S. Eurasian Chemicals PVT. Ltd. and kb-NB142-70 from Tocris Bioscience (UK). VEGF_165_ was purchased from Calbiochem (Merck-Millipore, Germany). Antibodies against phospho-Ser744/748 PKD1, phospho-Ser916 PKD1, total PKD1, phospho-Tyr951 VEGFR2, total VEGFR2, and *β*-actin were from Cell Signaling Technology. Phospho-Ser498 HDAC5 and total HDAC5 antibodies were from Santa Cruz Biotechnology. Phorbol 12-myristate 13-acetate (PMA) and bovine serum albumin (BSA) were purchased from Sigma-Aldrich.

### Recombinant kinase assay

Recombinant PKD1 (Lot: SP004) and VEGFR2 (Lot: 014) kinases were purchased from ProQinase GmbH (Germany). PKD reaction buffer contained 20 mM HEPES (pH 8.0), 1 mM DTT, 0.4 mM MnCl_2_, 0.01% (v/v) Brij-35, 0.41 μM ATP (K_M[ATP]_ value) and TAMRA-GS-derived peptide (5TAMRA-KKLNRTLSVA-OH, Molecular Devices) as substrate. The final PKD1 concentration was 20 nM. The enzyme reaction was incubated for 30 min and stopped by addition of 10 μl IMAP detection mixture (100% (v/v) IMAP Binding Buffer A and 1/400 IMAP Binding reagent, Molecular Devices). VEGFR2 reaction buffer contained 20 mM HEPES (pH 7.5), 1 mM DTT, 10 mM MgCl_2_, 0.01% (v/v) Tween-20, 32 μM ATP (K_M[ATP]_ value) and Poly Glu-Tyr (4:1) peptide as substrate at 0.04 mg/ml. The final VEGFR2 concentration was 6 nM. Reaction time was 1 hour at room temperature. Detection of the produced ADP was performed by Transcreener assay (Bellbrook Labs) according to the manufacturer’s instructions. Measurements were performed on an Analyst GT multimode reader. IC_50_ values were calculated using Excel (Microsoft) and XLfit (IDBS) softwares. Each measurement was repeated at least three times.

### Cell culture

The immortalized endothelial EA.hy926 cells, obtained from ATCC (ATCC CRL-2922), were grown in DMEM (Lonza) supplemented with 10% (v/v) FBS (Lonza) and 1% (v/v) antibiotic mix (MycoZap Plus-CL, Lonza) in humidified atmosphere of 37°C and 5% CO_2_. The RBL-2H3 cell line was obtained from Dr. R. Siraganian (National Institutes of Health, Bethesda MD), the depositor of this cell line at ATCC (ATCC CRL-2256). RBL-2H3 cells were, maintained in DMEM supplemented with 5% (v/v) FBS, 2 mM glutamine and antibiotics in a humidified atmosphere of 5% CO_2_ at 37°C. Tissue culture media and supplements used for the RBL-2H3 cell line were purchased from Gibco (Grand Island, NY).

### Cell viability assay

EA.hy926 cells were seeded into 96 well plates at a density of 10^4^ cells/well and were treated with the compounds in complete medium. After treatment, medium was removed and 30 μl serum-free DMEM and 20 μl PBS containing 5 mg/ml [3-(4,5-dimethylthiaziazol-2-yl)-2,5-diphenyl-2*H*-tetrazolium bromide] (MTT) was added and cells were incubated for 2 hours in 37°C. After the incubation tetrazolium crystals were dissolved in isopropanol containing 10% (v/v) Triton X-100 and 1% (v/v) 0.1 N HCl. Absorbance was measured at 570 nm and 690 nm with a Synergy multimode reader (BioTek). The 690 nm data was subtracted from the 570 nm for each well. Absolute IC_50_ values were calculated by non-linear regression using Graph Pad Prism 5 software. Each experiment was repeated at least three times.

### Cell proliferation assay

The proliferation of EA.hy926 cells was determined by the direct counting of the cells after 0.4% Trypan Blue solution (Sigma-Aldrich) staining in 1:1 ratio. Cell number was counted by Countess cell counter (Invitrogen).

### Western blot

EA.hy926 cells were grown in 6 cm Petri dishes to 90–95% confluence. Prior to treatment cells were starved in serum and antibiotic-free DMEM for 2–4 hours, and then were treated with the compounds in serum and antibiotic-free DMEM medium. After the treatment, cells were washed twice with ice-cold PBS and lysed in RIPA buffer (50 mM Tris (pH 7.4), 150 mM NaCl, 1% (v/v) NP-40, 0.5% (m/v) sodium-deoxycholate, 0.1% (m/v) sodium dodecyl sulphate, 2 mM EDTA, 2 mM EGTA, 50 mM NaF, 1 mM dithiothreitol, 1mM sodium-ortovanadate and protease inhibitor cocktail (Calbiochem)) for 30 minutes on ice. Lysates were centrifuged with 10 000 g at 4°C for 15 minutes. Equal amounts of protein (20–80 μg) were subjected to SDS-PAGE and electrotransferred to polyvinylidene-difluoride (PVDF) membranes. Membranes were probed with a primary antibody at 4°C overnight, and with horse radish peroxidase (HRP) conjugated secondary antibody for 1 h at room temperature. Bands were visualized by Enhanced Chemiluminescence (ECL) detection system (Perkin Elmer) and quantified by ImageJ v1.48 software.

### Wound healing assay

EA.hy926 cells were grown to 95% confluence in 3 cm Petri-dishes and then starved in serum-free DMEM overnight before scratching. The scratching was performed with a 200 μl pipette tip, then cultures were washed 3 times with PBS and compounds were added in full DMEM medium. Images were taken using an inverted phase-contrast microscope applying 10x objective (Alpha-Optika XDS). Quantification was made by ImageJ v1.48 software.

### Tube Formation

50 μl of ice-cold Matrigel from the same lot (BD Biosciences) was added to a 96-well plate and was let to polymerize at 37°C for 30 minutes. 1.5x10^4^ EA.hy926 cells were seeded into each well with or without the compounds in serum and antibiotic-free DMEM medium. Photos of the representative areas were taken by inverted phase-contrast microscope with 10x objective and quantification, based on 4 independent experiments, was made by ImageJ v1.48 Angiogenesis Analyzer Plugin.

### Neutrophil isolation, inhibitor treatment and activation

Human neutrophil isolation and superoxide production assay was carried out as described previously [29]. The human neutrophil studies were performed with the permission of Semmelweis University Regional and Institutional Committee of Science and Research Ethics (173/2006.)

### Secretory response of mast cells

*p*-nitrophenyl-*N*-acetyl-β-D-glucosamine was purchased from Sigma-Aldrich. 2,4-dinitrobenzene sulphonic acid-conjugated bovine serum albumin (DNP_11_-BSA) and murine DNP-specific monoclonal A2 IgE were kindly donated by Mr. Arieh Licht (Rehovot, Israel). For the experiments cells were detached by 15 min incubation with 10 mM EDTA in DMEM. Mediator secretion by mast cells in response to stimulation by FcεRI clustering was monitored by measuring activity of the secreted granular enzyme β-hexosaminidase, as described earlier [30]. To study the effect of VCC251801 on antigen-induced response, mast cells sensitized with DNP-specific A2 IgE were seeded in 96 well plate at a density of 10 000 cells/well and allowed to attach for 2 hours. Adherent cells were then preincubated with different concentrations of VCC251801 for 10 min at 37°C before exposure to suboptimal (10 ng/ml) antigen concentrations for 45 min at 37°C.

### Statistical analysis

Every experiment was performed at least three times. Statistical analysis was completed using Graph Pad Prism 5 software. The data of recombinant kinase assay, western blot analysis and tube formation is presented as mean±SD and data of viability assay, wound healing assay, neutrophil respiratory burst and mast cell degranulation assay is presented in mean±SEM. For statistical conparsion of the groups one way ANOVA-test, which followed by post hoc Dunett's Multiple Comparison Test was used. *p* value of < 0.05 was considered statistically significant.

## Results

### The identification of VCC251801, a potent inhibitor of VEGFR2 and PKD1

**Fig 1:**
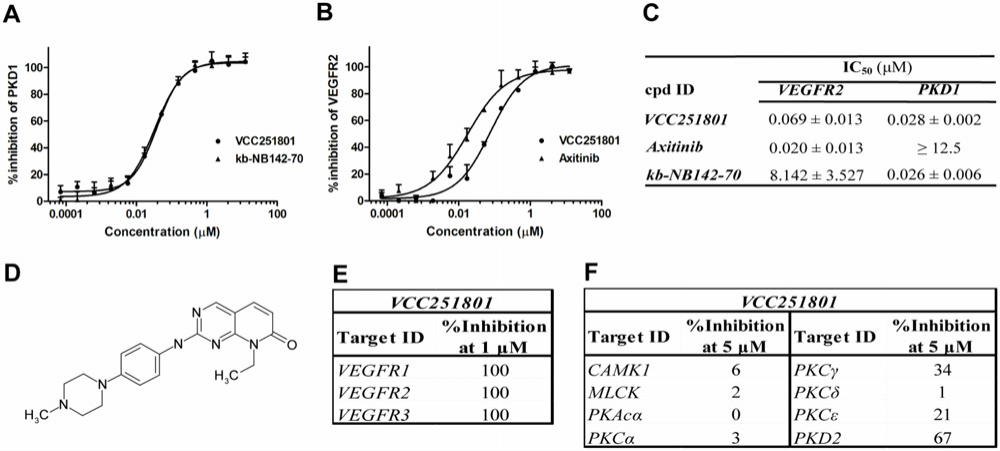
VCC251801 was an effective inhibitor of VEGFR2 and PKD1 in recombinant kinase assays. The dose response curves of VCC251801 in recombinant VEGFR2 **(A)** and PKD1 assays **(B)**. VEGFR2 inhibition was determined by Transcreener assay and PKD1 inhibition was determined applying IMAP assay. Axitinib was used as VEGFR2 reference compound and kb-NB142-70 as PKD1 reference compound. **(C)** The biochemical IC_50_ values of the inhibitors (mean ± SD) were calculated from at least three independent experiments. **(D)** The chemical structure of VCC251801. **(E)** Recombinant kinase assay against VEGFR isoforms was performed by ProQinase GmbH at single 1 μM concentration. **(F)** Selectivity study was carried out by SignalChem Ltd. at single 5 μM concentration.

The Vichem Chemie Ltd’s Extended Validation Library (EVL) was tested against recombinant PKD1 enzyme at a single concentration of 10 μM. The EVL contains more than 2000 known small molecule kinase inhibitor compounds and several in-house developed molecules based on 108 core structures [31]. Compounds showing more than 75% inhibition were selected and their biochemical IC_50_ values were determined for VEGFR2 and PKD1, using recombinant kinase assays. We identified the pyrido[2,3-*d*]pyrimidin-7-one core structure based VCC251801 as our best compound with potent VEGFR2 (IC_50_: 69 nM) and PKD1 (IC_50_: 28 nM) inhibitory effect (Fig 1A–1D). In our experiments we applied reference inhibitors against VEGFR2 and PKD1 such as Axitinib and kb-NB142-70, respectively. The IC_50_ value of Axitinib was 20 nM in VEGFR2 assay and it had no significant effect on PKD1, the IC_50_ value of kb-NB142-70 was 26 nM in PKD1 assay and it did not inhibit VEGFR2. VCC251801 was previously described [32] as an inhibitor of cyclin-dependent kinase 2 and 4 (CDK2/4), which was verified by previous *in vitro* kinase assays in our laboratory (data not shown). The compound was further investigated in selectivity study against VEGFR isoforms, different relevant kinases of the VEGFR2 signaling pathway and kinases showing high sequence or functional similarities with PKD1 (Fig 1E–1F). In this selectivity study, VCC251801 blocked both VEGFR1 and VEGFR3 activation, furthermore it showed moderate inhibitory effect against the other PKD isoform, PKD2. However, VCC251801 did not inhibit significantly the classical α and γ, or the novel δ and ε PKC-isoforms and it had no significant inhibitory effect on CAMK1 and MLCK enzymes, which kinases show high sequence homology with the catalytic domain of PKD enzymes. These data clearly show that VCC251801 effectively inhibited both VEGFR2 and PKD1 activity in recombinant kinase assays.

### VCC251801 did not alter cell viability significantly

**Fig 2:**
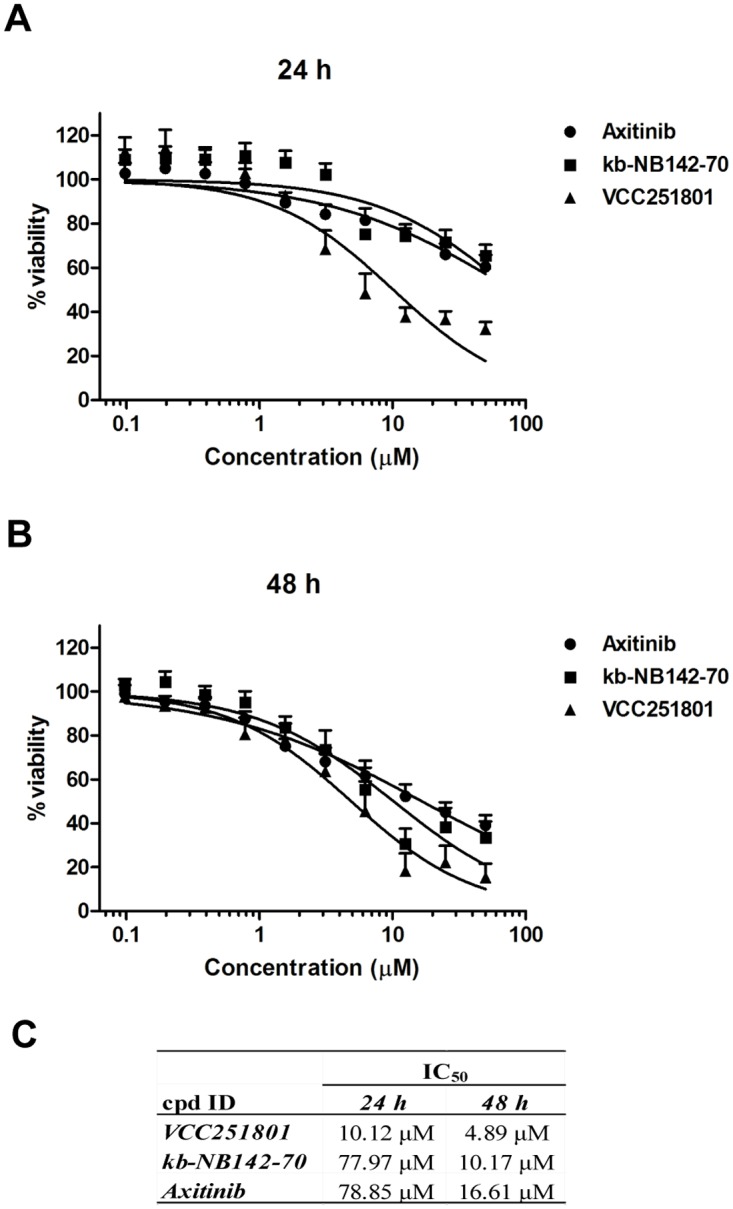
Treating endothelial cell with VCC251801 did not interfere with cell viability. The endothelial EA.hy926 cells were incubated with VCC251801 and reference compounds in 0.4–50 μM concentration range for 24 **(A)** and for 48 **(B)** hours at constant 0.5% DMSO concentration and cell viability was determined by MTT-assay. **(C)** Absolute IC_50_ values from MTT-assays were calculated using non-linear regression from at least three independent experiments.

Since VEGFR2 and PKD1 related pathways have a proven role in angiogenesis and in inflammation, we chose different angiogenesis and inflammatory cell models to further characterize the inhibitor. At first, we tested the effect of VCC251801 on cell viability and its potential cytotoxicity in the HUVEC-derived, immortalized EA.hy926 endothelial cell line. EA.hy926 cell line, established by Edgell et al. is a permanent cell line, showing endothelial properties, therefore it is a good model system to investigate *in vitro* angiogenesis [33–35]. EA.hy926 cells were treated with increasing doses of the inhibitors for 24 and 48 hours and cell viability was determined by MTT-assay. The IC_50_ value of VCC251801 was 10.12 μM after 24 hours and 4.89 μM after 48 hours treatment. The reference compounds Axitinib and kb-NB142-70 had even higher IC_50_ values in this viability assay (Fig 2A–2C). We also measured cell proliferation by counting viable cells staining with Trypan Blue after treatment with compounds and got also relatively high IC_50_ values (S1 Fig). Thus, the investigated kinase inhibitors only partially changed endothelial cell viability and proliferation, which strongly supports the idea that none of them interfered with viability in any of our experiments at the concentrations and time-spans used.

### VCC251801 inhibits VEGFR2 and PKD1 related signaling pathways in endothelial cells

**Fig 3:**
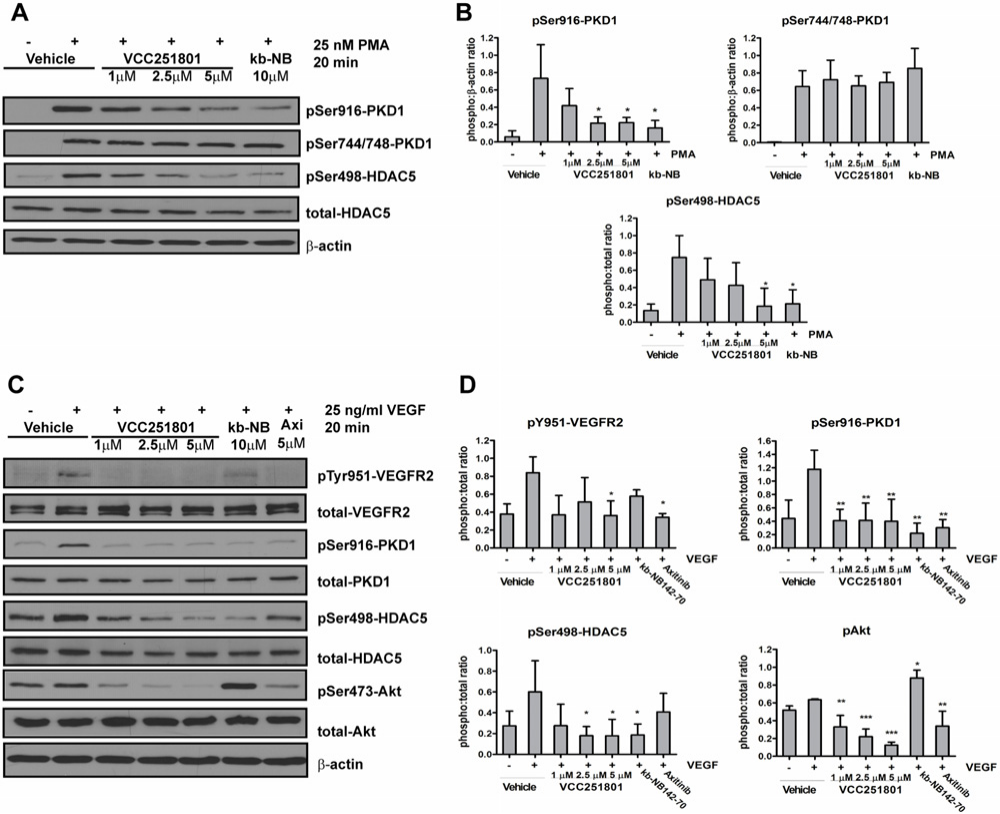
VCC251801 inhibited VEGFR2 and PKD1 related signaling pathway in endothelial cells. EA.hy926 cells were pre-treated with the inhibitors for 1 hour followed by the stimulation of 25 nM PMA, then PKD1 activity was analyzed (**A-B**). Next, 25 ng/ml VEGF activation was used after 1 hour pre-treatment of the inhibitors and VEGFR2 pathway involving PKD1 was monitored (**C-D**). Every experiment was carried out at least 3 times at constant 0.2% DMSO concentration; *, p < 0.05; **, p < 0.01; ***, p < 0.001.

In the further studies, we investigated the intracellular inhibitory effect of VCC251801 in the EA.hy926 cell line. First, we confirmed the PKD1 inhibitory effect of VCC251801 besides VEGFR2. We used the diacyl-glicerol (DAG) analogue phorbol 12-myristate 13-acetate (PMA), which activates every DAG sensitive kinase, particularly PKCs and also PKD isoforms in a PKC dependent manner [36,37]. PMA induces robust and non-specific kinase activation without the upstream activation of VEGFR2. After a short exposure to PMA, phosphorylation occurred at the PKD1 trans-phosphorylation site (Ser744/748) by PKC isoforms and at the C-terminal autophosphorylation site (Ser916). Since the Ser916 phosphorylation is not an unambiguous marker of PKD1 activity we monitored the phosphorylation level of the endogenous PKD1 substrate HDAC5 as well, where also a rapid increase was observed by the stimulation of PMA [38]. Pre-treatment with VCC251801 significantly decreased PMA-induced phosphorylation at PKD1 Ser916 and at HDAC5 Ser498 in a concentration dependent manner. PKD1 reference compound kb-NB142-70 also reduced PKD1 activity at 10 μM. However the phosphorylation of Ser744/748 remained unaffected in both cases (Fig 3A and 3B).

Next, we focused on the effect of VCC251801 on VEGFR2 and on VEGFR2 signaling pathway. The stimulation of EA.hy926 cells by VEGF caused a rapid increase in the VEGFR2 Tyr951 phosphorylation, which is important for the downstream activation of PKD1 [17,39]. The phosphorylation of PKD1 and its endogenous substrate HDAC5 on Ser498 was also increased by VEGF treatment. The pre-treatment of cells with the VEGFR2 inhibitor Axitinib or VCC251801 significantly reduced the VEGFR2 Tyr951 phosphorylation whereas the PKD inhibitor kb-NB142-70 had no significant effect on it. The reference compounds and VCC251801 significantly decreased PKD1 autophosphorylation (Ser916). However, PKD1 substrate HDAC5 Ser498 residue phosphorylation was only diminished by kb-NB142-70 and VCC251801. We also investigated Akt phosphorylation, which plays an important role in the progression of angiogenesis and endothelial cell survival. Axitinib and VCC251801 markedly inhibited Akt Ser473 residue phosphorylation however kb-NB142-70 even enhanced it (Fig 3C and 3D). In all, western blot analysis showed that VCC251801 could efficiently block VEGFR2 signaling pathway in endothelial cells.

### VCC251801 reduced endothelial cell migration and angiogenesis *in vitro*

**Fig 4:**
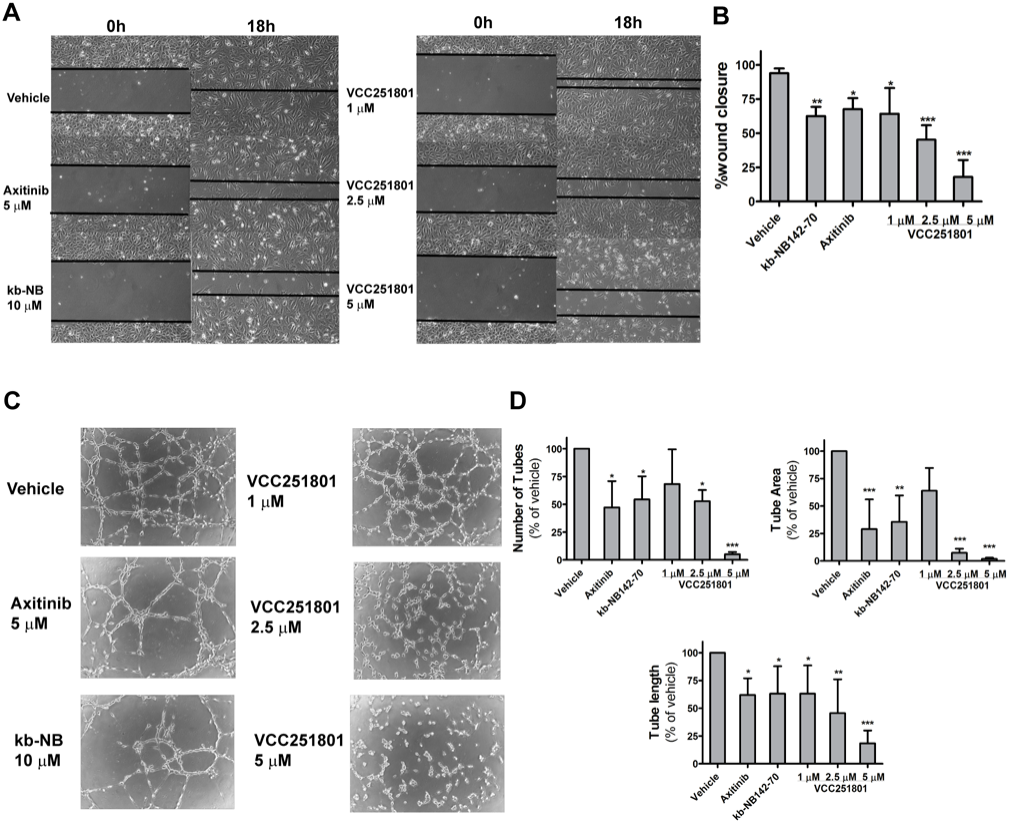
VCC251801 reduced endothelial cell migration and *in vitro* angiogenesis. Applying wound healing-assay, confluent EA.hy926 cell layer was scraped and were treated with the indicated inhibitors. Photos were taken at start point (0 h) and after 18 hours. Two photos were taken of each well in each experiment (n = 4) (**A-B**) Using tube formation assay, EA.hy926 cells were placed on Matrigel and tube formation was assessed and photographed after 18 hours **(C)** The number of the capillary-like tubes, tube area and tube length were determined using ImageJ Agiogenesis Analyzer Plugin from 3 independent experiment (**D**). Every treatment was carried out at constant 0.2% DMSO concentration; *, p < 0.05; **, p < 0.01; ***, p < 0.001.

Endothelial cell migration plays an essential role in the first steps of angiogenesis. To investigate the anti-migratory effect of VCC251801 we used *in vitro* wound healing or scratch assay. Serum-induced EA.hy926 cells almost completely covered the wound after 18 hours (98%). The reference compounds Axitinib (5 μM) and kb-NB142-70 (10 μM) reduced endothelial cell migration by 32% and 38%, respectively. VCC251801 caused even higher reduction of wound closure: 35% at 1 μM, 55% at 2.5 μM and 82% at 5 μM treatment (Fig 4A and 4B).

Tube formation assay is based on the phenomenon that endothelial cells placed on extracellular matrix are able to form capillary-like tube structures, hence this is a good model to investigate angiogenesis *in vitro*. At first in this process, endothelial cells begin to show projections called sprouting and migrate toward each other to establish cell-cell connections. These multiple cell-cell connections are able to form polygon structures, which can turn into tubules with walls, made up of 2 or 3 cells, called complex mesh [40]. In this assay, Axitinib (5 μM) showed 47% tube number, 70% tube area and 40% tube length reduction. Treatment the cells with kb-NB142-70 (10 μM) resulted in 53% tube number, 65% tube area and 37% tube length reduction. VCC251801 caused more significant reduction of tube number by 47% tube area by 93% and tube length by 55% already at 2.5 μM. The effect was more significant at 5 μM because tube number was reduced by 95%, tube area by 98% and tube length by 82%. However, the effect of VCC251801 was not significant at 1 μM concentration, except the tube number decrease, where the reduction was 37% (Fig 4C and 4D). In conclusion, VCC251801 was proved to be an effective inhibitor of *in vitro* angiogenesis.

### VCC251801 effectively inhibited neutrophil superoxide production

**Fig 5:**
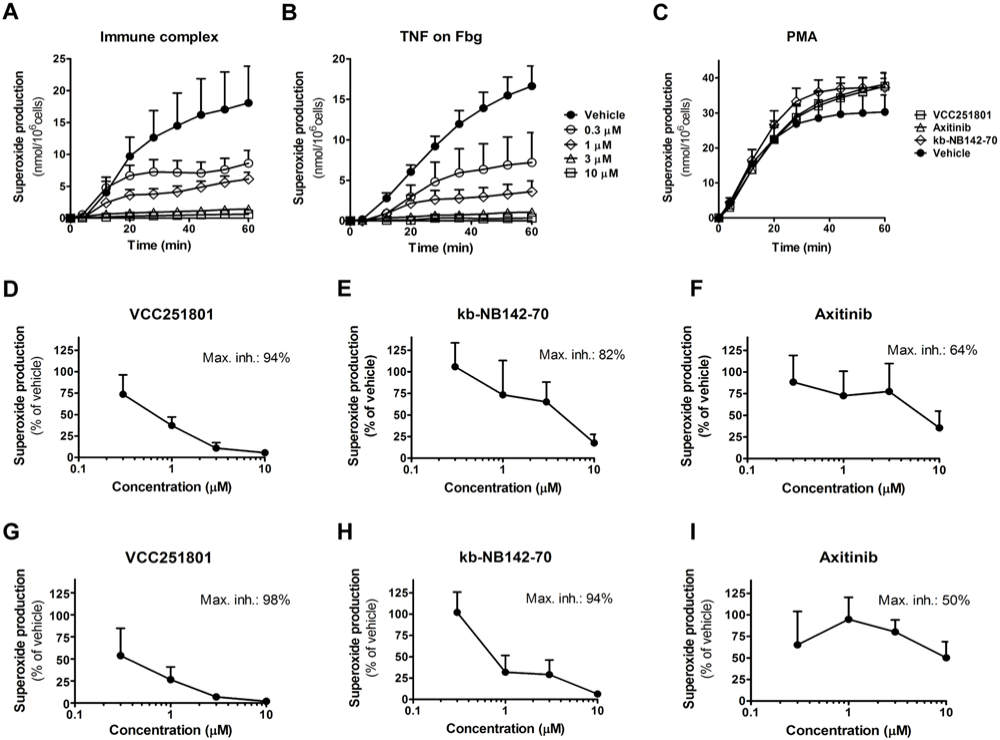
VCC251801 specifically inhibited neutrophil response. Human neutrophils were pre-treated with VCC251801 for 30 minutes in 37°C at the following concentrations: 0.3 μM, 1 μM, 3 μM, 10 μM. Respiratory burst was induced by immune-complex **(A)** or 20 ng/ml TNFα on Fibrinogen coating **(B)**. Every compound was tested at 10 μM using 100 nM PMA stimulation **(C)**. The dose response curves of VCC251801 and the reference inhibitors are presented using immune complex **(D-F)** or TNFα on fibrinogen coating **(G-I)** stimulation. In dose response experiments, each compound was used at the concentrations mentioned above. Every experiment was performed at least 3 times at constant 0.1% DMSO concentration.

Polymorphonuclear leucocytes or neutrophils are differentiated phagocytic cells, involved in the defense against bacterial or fungal pathogens and in the development of various acute and chronic inflammatory diseases. Recent data show that PKD1 is involved in the immune complex mediated neutrophil cell response [21,41]. One can follow neutrophil activation by various cell responses, in our functional model we measured the production of reactive oxygen species generated by NADPH oxidase enzymes. For neutrophil activation we used three different methods: (1) activation by immune-complex (IC) through Fc-receptors, (2) adherent activation by Fibrinogen and TNFα and (3) an unspecific activation by the previously used diacylglycerole analogue phorbol-ester PMA (Fig 5A–5C). The first two types of activation are physiological stimulations and they have a pivotal role in autoimmune diseases like in rheumatoid arthritis. However, PMA induces robust, unspecific, universal kinase and neutrophil activation. The immune-complex induced superoxide production of neutrophils was almost completely blocked by VCC251801 already at 3 μM concentration (89% inhibition) (Fig 5A and 5D). kb-NB142-70 also reduced superoxide production by 82% at 10 μM (Fig 5E), however Axitinib caused only 62% decrease in cell response even at the highest 10 μM dose (Fig 5F). The TNF-α induced neutrophil activation was also completely blocked by VCC251801 at 3 μM treatment (93%) and the maximal inhibition was 98% at 10 μM (Fig 5B and 5G). It was markedly better than PKD1 reference compound kb-NB142-70 where 10 μM treatment was needed to achieve the same inhibitory effect (Fig 5H). We observed similar results using Axitinib, which could cause only 50% inhibition even at 10 μM treatment (Fig 5I). None of the used inhibitors could interfere with PMA induced neutrophil response even at 10 μM dose (Fig 5C). According to these data, VCC251801 was able to abrogate neutrophil cell response to specific stimuli.

### VCC251801 reduced antigen-induced mediator release from mast cells

**Fig 6:**
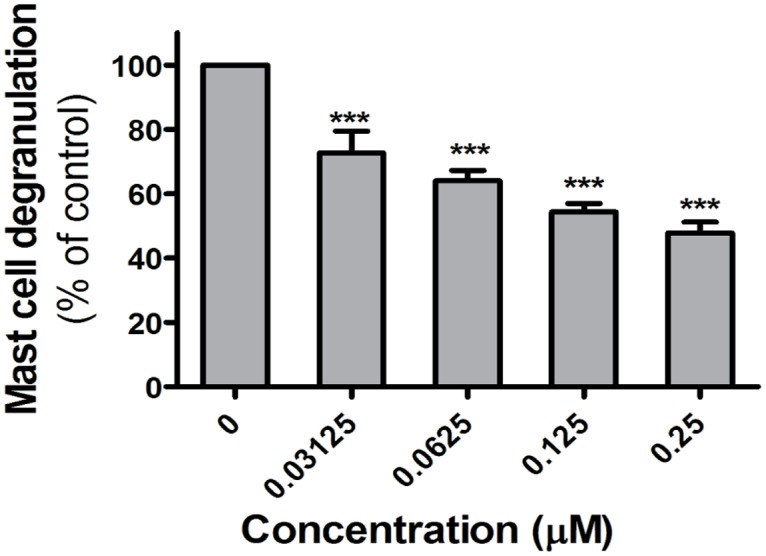
The FcεRI triggered response of mast cells was reduced by VCC251801. Inhibition of antigen-induced mediator release from Adherent RBL-2H3 mast cells were treated with VCC251801 for 10 minutes then activated by 10 ng/ml antigen and cell degranulation was measured; ***, p < 0.001.

Mast cells are known to play an important role in many inflammatory responses [42]. Clustering of the high affinity FcεRI on their plasma membrane triggers these cells to secrete inflammatory mediators, including histamine and various enzymes. PKD1 has a proven role in the FcεRI-induced cell response of mast cells [23]. In our experimental model system the rat RBL-2H3 mast cell line was used to investigate the effect of VCC251801. RBL-2H3 cells were sensitized with antigen specific IgE, followed by incubation with the indicated concentrations of VCC251801 for 10 minutes. Clustering of FceRI was induced by DNP, and activation of the cells was assessed by measuring the release of the granular enzyme, beta-hexoseaminidase. As shown in Fig 6, VCC251801 inhibited dose dependently the degranulation of mast cells.

These experiments show that VCC251801 efficiently reduced FcεRI triggered activation of mast cells.

## Discussion

During chronic inflammation, inflammatory cells and factors are able to promote pathologic angiogenesis, induce extensive tissue damage and even foster carcinogenesis. The major regulator of angiogenesis is the VEGFR2 signaling pathway, which also has an important role in inflammatory processes [1,14]. PKD1 as one of the members of the VEGFR2 signaling axis has an important role in cell proliferation, angiogenesis and in inflammation [19,22]. In addition, PKD1 is involved in the superoxide production of neutrophils and in mediating mast cell degranulation [21,23,43]. Although potent VEGFR2 inhibitors are currently available (such as the pan VEGFR inhibitor Axitinib, approved to treat renal cell carcinoma [44]), it is highly possible that using single target inhibitors secondary resistance will occur. That is why the use of multiple target inhibitors would be more advantageous [45].

Unfortunately, the best available PKD1 inhibitor (only available for basic research), kb-NB142-70 still has many weaknesses [46]. Thus, due to the currently available limited therapeutic interventions a potent multiple target kinase pathway inhibitor would be of a great value e.g. for the treatment of pathological angiogenesis and inflammation related diseases.

In this study we identified and characterized a multiple kinase inhibitor VCC251801 which effectively inhibited the VEGFR2—PKD1 axis in pathological angiogenesis and inflammation related processes. VCC251801 is based on a pyrido[2,3-*d*]pyrimidin-7-one core structure and was previously identified as a CDK 2/4 inhibitor by Barvian et al. [32], in accordance with our previous observations (data not shown). We compared the VEGFR2 and PKD1 inhibition of VCC251801 to reference VEGFR2 (Axitinib) and PKD1 (kb-NB142-70) inhibitor compounds. The IC_50_ value of VEGFR2 reference compound Axitinib was 20 nM in *in vitro* recombinant kinase assay, which is higher than the IC_50_ value measured by Hu-Lowe et al. (0.2 nM) [47]. The reason of the difference might be the applying of different assay methods. Despite this difference Axitinib proved to be an effective inhibitor against VEGFR2 in our system as well. Regarding PKD1 inhibition, we measured very similar IC_50_ value of kb-NB142-70 (26 nM) to the data published by Lavalle et al. [48].

VCC251801 inhibited all of the three VEGFR isoforms in our selectivity study, which is not surprising because most known VEGFR inhibitors are not isoform specific [49]. The currently available PKD1 inhibitors also have strong inhibitory effect also on PKD2 or 3 isoforms [48,50,51]. However, VCC251801 showed only moderate effect on PKD2 which might help to develop PKD isoform specific small-molecule inhibitors based on structure-activity relationship data. VCC251801 proved to be selective for PKD1, and did not inhibit other kinases with high catalytic sequence homology (CAMK or MLCK), or functionally similar PKC isoforms. Although VCC251801 blocked the activity of the three VEGFR isoforms *in vitro*, in the further experiments we focused only on the VEGFR2 signaling pathway, because VEGFR2 is the dominant regulator and the most validated target of angiogenesis [4]. Furthermore, VEGFR2 is implicated in several inflammatory diseases. Since PKD1 is also a validated target in angiogenesis and inflammation-related diseases, we further characterized VCC251801 in inflammatory cellular models.

In EA.hy926 endothelial cells VCC251801 only a slightly changed the cell viability not interfering with the effects of our compounds in the further experiments with the applied conditions. Monitoring the intracellular effect of the inhibitor using western blot analysis, VCC251801 was indeed found to be an effective inhibitor of PMA-induced endogenous PKD1 activation. PMA activates every DAG sensitive kinases including PKC isoforms and PKD1 through PKC activation bypassing upstream activation-for example by VEGF. This experiment verifies the selectivity study as well, namely VCC251801 had no inhibitory effect on the PKD1 activating upstream PKC isoforms such as β, δ or ε, because the trans-phosphorylation level (Ser744/748) did not change by the pre-treatment of the inhibitor. VCC251801 was also an efficient inhibitor of VEGFR2 and its signaling pathway. Investigating VEGFR2 signaling pathway in endothelial cell line, VCC251801 greatly decreased the phosphorylation of HDAC5 in contrast to the minor effect of the VEGFR2 inhibitor Axitinib. Moreover the VEGFR2 signaling pathway inhibition was further verified by analyzing Akt phosphorylation level which has crucial role in cell survival. Previous study by Ni et al. indicated that the inhibition of PKD1 in GPCR pathways resulted in enhanced Akt activation, due to the negative feedback between PKD1 and PI3K/Akt kinases. In accordance with this study we showed that blocking PKD1 by kb-NB142-70 enhanced Akt phosphorylation also in the receptor tyrosine kinase VEGFR pathway in endothelial cells [52]. However, Axitinib and VCC251801 due to their VEGFR2 and multiple kinase inhibitory effect significantly diminished Akt kinase phosphorylation. This could be an important fact in development of PKD inhibitors for clinical use, because the single inhibition of neither VEGFR2 nor PKD1 is not sufficient, but targeting both can block HDAC5 and Akt activity, as well. Targeting VEGFR2, PKD1 and other relevant kinases could be an advantage in therapeutic application, when resistance evolves against VEGFR2 inhibitors through the activation of alternative signaling pathways.

In the further experiments, we investigated the effect of VCC251801 on different cell functions in inflammatory cell models. The endothelial EA.hy926 cell line was applied for wound healing and tube formation assays to model angiogenesis *in vitro* wherein the VEGFR2 signaling pathway plays a dominant role. In the beginning of angiogenesis, endothelial cells migrate toward to the location of chemoattractants secreted by inflammatory cells or malignant tumors. Following cell migration by wound healing assay, VCC251801 was more effective (even in low concentrations) than any of the reference compounds, presumably again due to the simultaneous inhibition of VEGFR2 and PKD1. Next we performed tube formation assay, which is based on the phenomenon that endothelial cells placed on extracellular matrix, compose capillary-like structures. The advantage of tube formation assay is that it involves many steps of angiogenesis (cell migration, proliferation and invasion) therefore it is a more realistic model of angiogenesis. In this assay, VCC251801 efficiently inhibited the formation of capillary-like tube structures in lower concentrations than the reference compounds, Axitinib and kb-NB142-70.

Furthermore, we investigated the inhibitory effect of VCC251801 on human neutrophil granulocytes. Neutrophils are differentiated phagocytic cells, involved in the defense against bacterial or fungal pathogens but also contribute to the development of various acute and chronic inflammatory diseases [41]. According to previous findings, neutrophils express PKD1, which has a positive regulatory role in Fcγ receptor-induced superoxide production by NADPH-oxidase enzymes [21]. In concordance with this study VCC251801 and kb-NB142-70 were effective inhibitors of the immune-complex stimulated superoxide production, confirming the regulatory role of PKD1 in this pathway. Interestingly in the same neutrophil assay both kb-NB142-70 and VCC251801 effectively inhibited TNFα induced respiratory burst on fibrinogen substrate, however the role of PKD1 is unknown in this pathway. These results might propose the involvement of PKD1 in this signaling pathway. In these experiments Axitinib was not a potent inhibitor of neutrophil cell response, probably due to the lack of any known functional role of VEGFR2 or the other VEGFR isoforms in superoxide production, although an important function of VEGFR2 in cellular chemotaxis is documented [53]. However, none of the inhibitors blocked PMA-induced neutrophil response, which suggests that VCC251801 was able to selectively block the signaling pathways involved in neutrophil superoxide production. Although, VCC251801 significantly abrogated neutrophil response to different stimuli, it was not able to reduce neutrophil transmigration induced by fMLP in transwell migration assay (S2 Fig). This result could correlate with previous studies by Ittner et al., because they showed that PKD1 has a negative regulatory role in mouse neutrophil infiltration to the lung [54].

The inhibitory effect of VCC251801 has been further verified in additional studies on mast cells, as other crucial players in autoimmune diseases [42]. Previous studies reported that in mast cells PKD1 is involved in the regulation of IgE induced cytokine release via FcεRI signaling [23,43,55]. In our study, treating mast cells with VCC251801 effectively reduced the FcεRI clustering induced degranulation, which could support the involvement of PKD1 inhibition by VCC251801. In our preliminary experiments carried out employing human basophils—another important cell type related to inflammation—VCC251801 also proved to be a potent inhibitor of cell activation (data not shown).

According to previous studies from our lab, in recombinant kinase assays VCC251801 did not inhibited significantly Src-family kinases, namely c-Src, Fgr, Lyn and Hck (the IC_50_ values of each kinases were higher than 3 μM), which kinases play pivotal role in several immune cell responses, e.g. in neutrophils and mast cells [56,57]. These data confirm the significance of our PKD1 signaling pathway inhibitory compound in neutrophil and mast cell system, exerting its effect without interfering with Src-kinase family enzymes.

Based on our results in various endothelial model systems and inflammatory cell types, we suggest that VCC251801 might be a potent pathway inhibitor of pathological angiogenic and inflammatory processes, targeting signaling pathways involving VEGFR2 and PKD1. Data in endothelial and inflammatory cells indicate that treatment with VCC251801 results in a blockade of VEGFR2 and PKD1 signal transduction. The strong *in vitro* inhibition of these inflammatory pathways could be a rationale for further in vivo studies in pathological inflammatory models.

## Supporting Information

S1 Fig The effect of VCC251801 on endothelial cell proliferation. The anti-proliferative effect of VCC251801 was determined by direct counting of viable cells staining with Trypan Blue after 24 **(A)** and 48 **(B)** hour treatment. **(C)** Absolute IC_50_ values were calculated using non-linear regression from at least three independent experiments.
(TIF)

S2 Fig None of the inhibitors affected neutrophil transwell migration. In this assay we used polycarbonate filters with 3 μm pore size, pre-coated with fibrinogen. Neutrophils were pre-incubated with the inhibitors at 37°C for 30 minutes, then were placed into the insert and were allowed to migrate toward 100 nM fMLP at 37°C for 1 hour (n = 3). The assay was performed as described in [29].
(TIF)
