# Correction: Targeting Vascular Endothelial Growth Factor Receptor 2 and Protein Kinase D1 Related Pathways by a Multiple Kinase Inhibitor in Angiogenesis and Inflammation Related Processes *In Vitro*

**DOI:** 10.1371/journal.pone.0144792

**Published:** 2015-12-16

**Authors:** Attila Varga, Pál Gyulavári, Zoltán Greff, Krisztina Futosi, Tamás Németh, Laura Simon-Szabó, Krisztina Kerekes, Csaba Szántai-Kis, Diána Brauswetter, Márton Kokas, Gábor Borbély, Anna Erdei, Attila Mócsai, György Kéri, Tibor Vántus

In the Funding section, the grant agreement name from the funder European Commission’s Seventh Framework Programme is listed incorrectly. The correct grant agreement name is as follows: The research leading to these results has received funding from the European Commission’s Seventh Framework Programme (FP7/2007-2013) under the grant agreement Lungtarget (project n° 259770).
