# Supplementary figures and images for: Targeting Vascular Endothelial Growth Factor Receptor 2 and Protein Kinase D1 Related Pathways by a Multiple Kinase Inhibitor in Angiogenesis and Inflammation Related Processes In Vitro
Source: PLoS One. 2015 Apr 14;10(4):e0124234. doi: 10.1371/journal.pone.0124234 (PMC4396990; doi:10.1371/journal.pone.0124234)

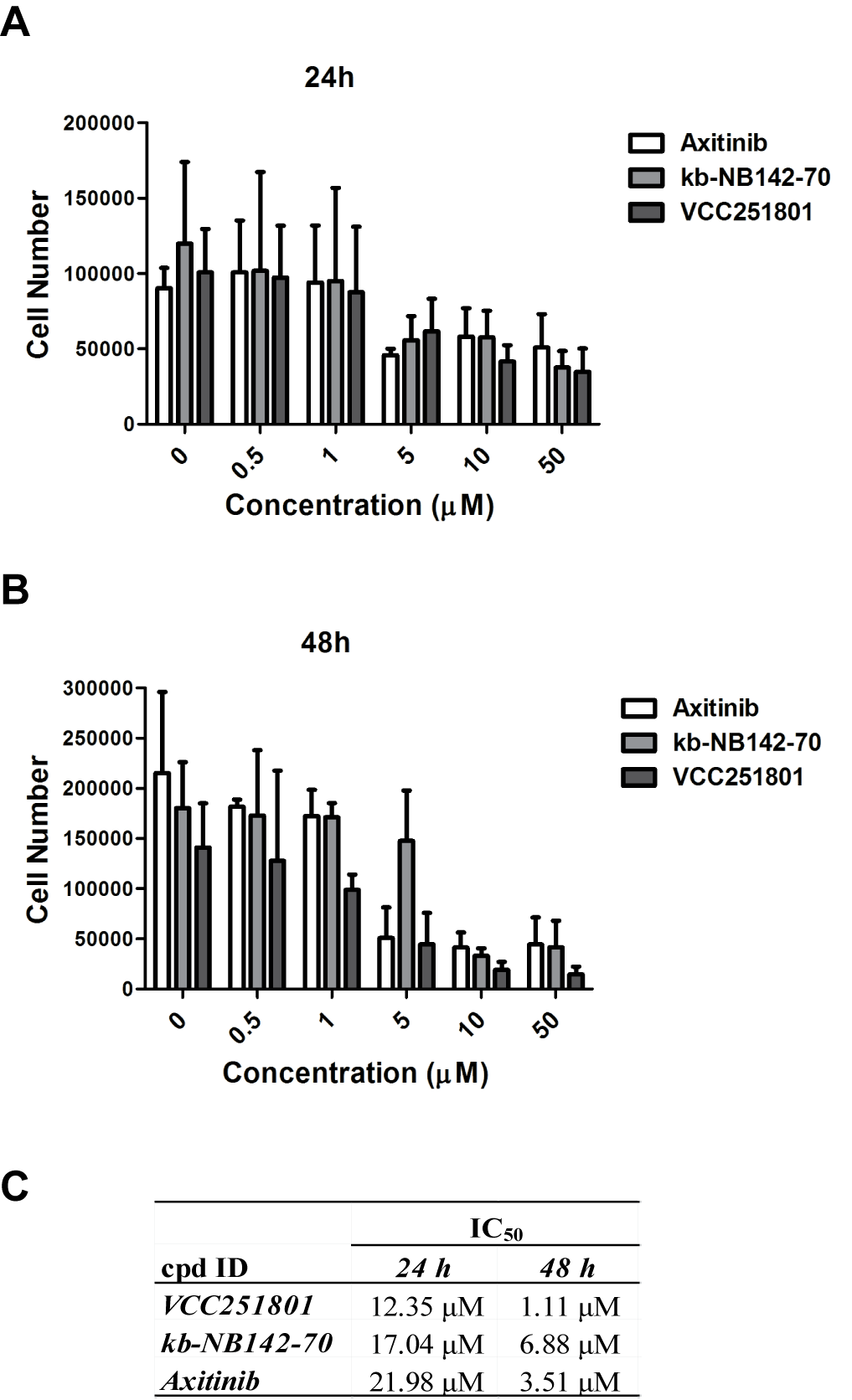

Supplement: S1 Fig — The anti-proliferative effect of VCC251801 was determined by direct counting of viable cells staining with Trypan Blue after 24 (A) and 48 (B) hour treatment. (C) Absolute IC50 values were calculated using non-linear regression from at least three independent experiments. (TIF) [file pone.0124234.s001.tif]

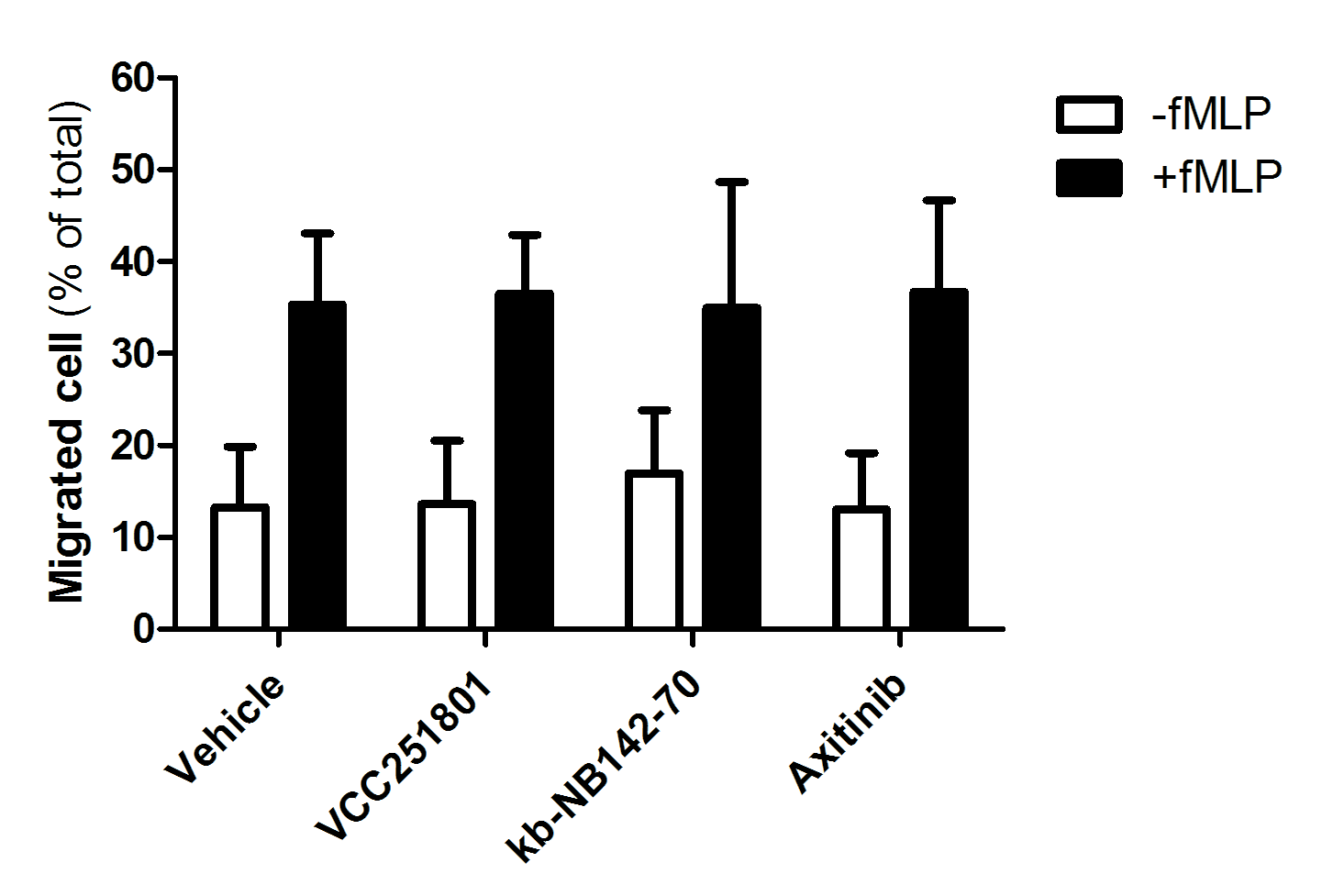

Supplement: S2 Fig — In this assay we used polycarbonate filters with 3 μm pore size, pre-coated with fibrinogen. Neutrophils were pre-incubated with the inhibitors at 37°C for 30 minutes, then were placed into the insert and were allowed to migrate toward 100 nM fMLP at 37°C for 1 hour (n = 3). The assay was performed as described in [29]. (TIF) [file pone.0124234.s002.tif]
